# Effects of Antifreeze Protein III on Sperm Cryopreservation of Pacific Abalone, *Haliotis discus hannai*

**DOI:** 10.3390/ijms22083917

**Published:** 2021-04-10

**Authors:** Shaharior Hossen, Md. Rajib Sharker, Yusin Cho, Zahid Parvez Sukhan, Kang Hee Kho

**Affiliations:** 1 Department of Fisheries Science, College of Fisheries and Ocean Sciences, Chonnam National University, 50 Daehak-ro, Yeosu 59626, Jeonnam, Korea; 186465@jnu.ac.kr (S.H.); mrsharker@pstu.ac.bd (M.R.S.); 198099@jnu.ac.kr (Y.C.); sukhan1026@jnu.ac.kr (Z.P.S.); 2 Department of Fisheries Biology and Genetics, Faculty of Fisheries, Patuakhali Science and Technology University, Patuakhali 8602, Bangladesh

**Keywords:** AFPIII, cryopreservation, post-thaw sperm quality, fertilization and hatching, mRNA expression of HSP90

## Abstract

Pacific abalone (*Haliotis discus hannai*) is a highly commercial seafood in Southeast Asia. The aim of the present study was to improve the sperm cryopreservation technique for this valuable species using an antifreeze protein III (AFPIII). Post-thaw sperm quality parameters including motility, acrosome integrity (AI), plasma membrane integrity (PMI), mitochondrial membrane potential (MMP), DNA integrity, fertility, hatchability, and mRNA abundance level of heat shock protein 90 (HSP90) were determined to ensure improvement of the cryopreservation technique. Post-thaw motility of sperm cryopreserved with AFPIII at 10 µg/mL combined with 8% dimethyl sulfoxide (DMSO) (61.3 ± 2.7%), 8% ethylene glycol (EG) (54.3 ± 3.3%), 6% propylene glycol (PG) (36.6 ± 2.6%), or 2% glycerol (GLY) (51.7 ± 3.0%) was significantly improved than that of sperm cryopreserved without AFPIII. Post-thaw motility of sperm cryopreserved with 2% MeOH and 1 µg/mL of AFPIII was also improved than that of sperm cryopreserved without AFPIII. A combination of 10 µg/mL AFPIII with 8% DMSO resulted in the highest post-thaw motility, showing AI of 60.1 ± 3.9%, PMI of 67.2 ± 4.0%, and MMP of 59.1 ± 4.3%. DNA integrity of sperm cryopreserved using 10 µg/mL AFPIII combined with 8% DMSO was not significantly (*p* > 0.05) different from that of fresh sperm. Cryopreservation using a combination of AFPIII with 8% DMSO improved fertilization and hatching rates of sperm compared to that of cryopreservation without supplementation of 10 µg/mL AFPIII. Sperm cryopreserved using AFPIII showed higher mRNA abundance levels of HSP90 than those cryopreserved without AFPIII. Results of the present study suggest that 10 µg/mL AFPIII combined with 8% DMSO can be used for large scale cryopreservation of Pacific abalone sperm and for hatchery production.

## 1. Introduction

The Pacific abalone, *Haliotis discus hannai,* is a seafood with a high commercial value in Korea, Japan, China, and Taiwan because it contains bioactive molecules that are beneficial to human health [1]. This species has been commercially cultured in Korea for the last three decades. In vitro reproduction of abalone offspring requires large amounts of sperm [2]. Progenitors of genetic high quality are obligatory for producing improved progenies [3]. Sperm cryopreservation methods are vital tools for obtaining high quality abalone progeny [4]. To date, sperm cryopreservation of *H. discus hannai* has been performed using penetrating cryoprotectants [2]. Although penetrating cryoprotectants can increase cell membrane fluidity through protein and lipid reorganization [5], they can also hamper intracellular ice crystal formation by decreasing the freezing point temperature, which is known to be an undesirable mechanism of sperm death [6,7]. The sperm cryopreservation process involves changes of extreme temperature that can lead to cryoinjuries (decreases of plasma membrane integrity, acrosomal integrity, mitochondrial membrane potential, motility, and viability) of morphological structures and physiology [8,9,10]. These cryoinjuries can alter the functional status of sperm proteins in the plasma membrane, midpiece, nucleus, and flagella, thus affecting the motility and fertilization capacity of sperm [11,12,13,14,15]. Nucleus DNA alteration of sperm may occur due to the freeze–thaw process of cryopreservation [16]. Sperm DNA integrity acts as one of the indicators of fertility potential. It can be assessed by comet assay [17], sperm chromatin structure assay (SCSA) [18], sperm chromatin dispersion (SCD) test [19], and TUNEL assay (terminal deoxynucleotidyl transferase-mediated dUDP nick end labelling) [20]. DNA fragmentation is known to reduce the fertilization capacity of sperm. It may lead to abnormal embryonic development [21].

The freeze–thaw process can also alter mRNA stability, epigenetic content, protein abundance levels, and mRNA transcript abundance levels of sperm [22]. Cryopreservation was shown to decrease mRNA abundance levels of heat shock protein 90 (HSP90) in oyster sperm [23] and bull sperm [24]. Kasimanickam et al. [25] reported that reduced mRNA abundance levels of sperm were associated with lower fertility of Holstein bulls. Reduced mRNA transcripts of cryopreserved sperm could affect molecular elements with an important role in fertilization success and correct early embryonic development [26].

Considering the above facts, the sperm cryopreservation protocol for this commercially valuable species needs to be improved. Sperm cryopreservation using antifreeze proteins (AFPs) can improve post-thaw sperm quality of different species of fish, including sea bream [27,28,29], sterlet [9,15], common carp [30], and Persian sturgeon [31]. AFPs are specific proteins that can protect cells by reducing the freezing point, interacting with the plasma membrane, adjusting ice crystallization, and protecting recrystallization [9,32,33,34]. A sperm cryopreservation method using AFP has not yet been reported for Pacific abalone sperm or aquatic invertebrates. Thus, the aim of the present study was to improve the sperm cryopreservation method for Pacific abalone using antifreeze protein. The specific aim of the present study was to improve post-thaw sperm motility, plasma membrane integrity (PMI), acrosome integrity (AI), mitochondrial membrane potential (MMP), DNA integrity, fertilization ability, and hatching capacity of Pacific abalone.

## 2. Results

### 2.1. Effects of AFPIII on Sperm Cryopreservation

#### 2.1.1. Combined Effects of AFPIII and DMSO on Post-Thaw Sperm Motility

**Figure 1:**
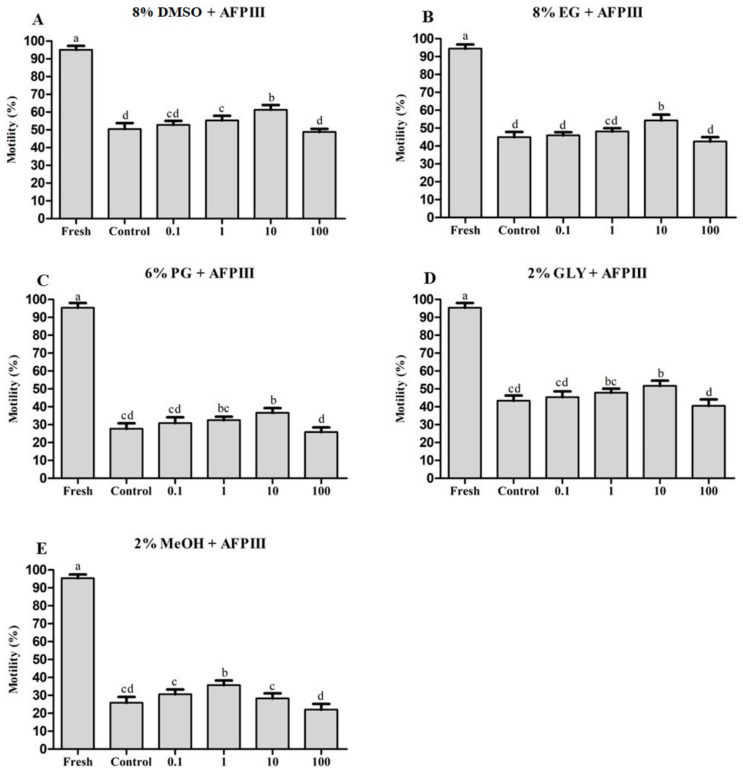
Post-thaw motility of sperm cryopreserved with antifreeze protein III (AFPIII) at different concentrations (0.1, 1, 10, and 100 µg/mL). Significantly different levels (*p* < 0.05) are denoted by different letters. (**A**) Combined effects of AFPIII with 8% dimethyl sulfoxide (DMSO) at different concentrations. (**B**) Combined effects of AFPIII with 8% ethylene glycol (EG) at different concentrations. (**C**) Combined effects of AFPIII with 6% propylene glycol (PG) at different concentrations. (**D**) Combined effects of AFPIII with 2% glycerol (GLY) at different concentrations. (**E**) Combined effects of AFPIII with 2% MeOH at different concentrations.

Sperm cryopreserved with 10 µg/mL AFPIII combined with 8% dimethyl sulfoxide (DMSO) showed the highest post-thaw motility (61.3 ± 2.7%) (Figure 1A). Their post-thaw motility was significantly (*p* < 0.05) improved than that of the control (8% DMSO).

#### 2.1.2. Combined Effects of AFPIII and EG on Post-Thaw Sperm Motility

AFPIII at 10 µg/mL combined with 8% ethylene glycol (EG) showed the highest post-thaw motility (54.3 ± 3.3%) (Figure 1B). This post-thaw motility was significantly (*p* < 0.05) higher than that of sperm cryopreserved with 8% EG alone (44.9 ± 2.9%).

#### 2.1.3. Combined Effects of AFPIII and PG on Post-Thaw Sperm Motility

Sperm cryopreserved using 10 µg/mL AFPIII combined with 6% propylene glycol (PG) showed the highest post-thaw motility (36.6 ± 2.6%) (Figure 1C). This post-thaw motility was significantly (*p* < 0.05) lower than the post-thaw motility of fresh sperm, but significantly higher than that of sperm cryopreserved with 6% PG alone without AFPIII.

#### 2.1.4. Combined Effects of AFPIII and GLY on Post-Thaw Sperm Motility

Sperm cryopreserved with AFPIII at 10 µg/mL combined with 2% glycerol (GLY) had the highest post-thaw motility (51.7 ± 3.0%) (Figure 1D). This post-thaw motility was significantly (*p* < 0.05) higher than that of sperm cryopreserved with 2% GLY without 10 µg/mL AFPIII (43.4 ± 2.9%). However, fresh sperm exhibited significantly (*p* < 0.05) higher motility (95.3 ± 2.7%) than that of all cryopreserved sperm.

#### 2.1.5. Combined Effects of AFPIII and MeOH on Post-Thaw Sperm Motility

Sperm cryopreserved with AFPIII at 1 µg/mL combined with 2% MeOH showed significantly (*p* < 0.05) higher post-thaw motility (30.7 ± 2.6%) than that of other cryopreserved sperm (Figure 1E). However, such post-thaw motility was significantly (*p* < 0.05) lower than the motility of fresh sperm.

### 2.2. Fluorescent Technique for Assessing PMI, AI, and MMP of Cryopreserved Sperm

#### 2.2.1. Plasma Membrane Integrity (PMI)

**Figure 2:**
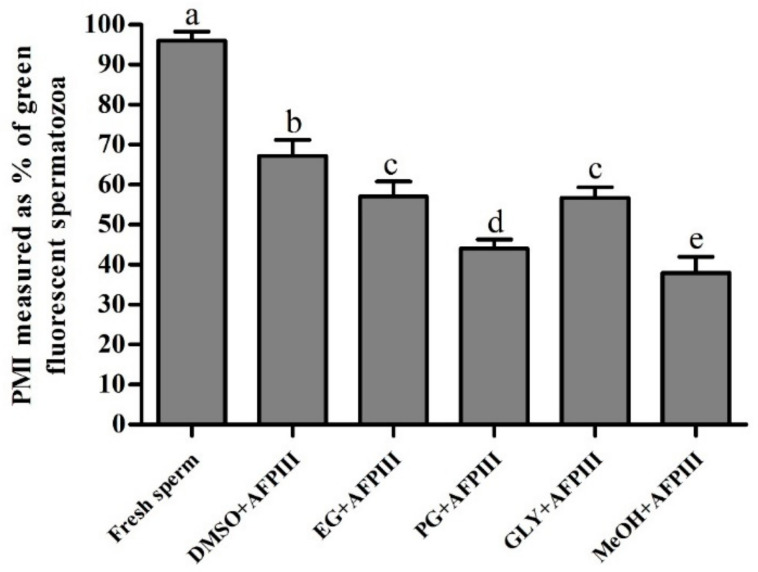
Plasma membrane integrity (PMI) (%) of post-thaw sperm of *H. discus hannai*. Significantly different levels (*p* < 0.05) are denoted by different letters.

**Figure 3:**
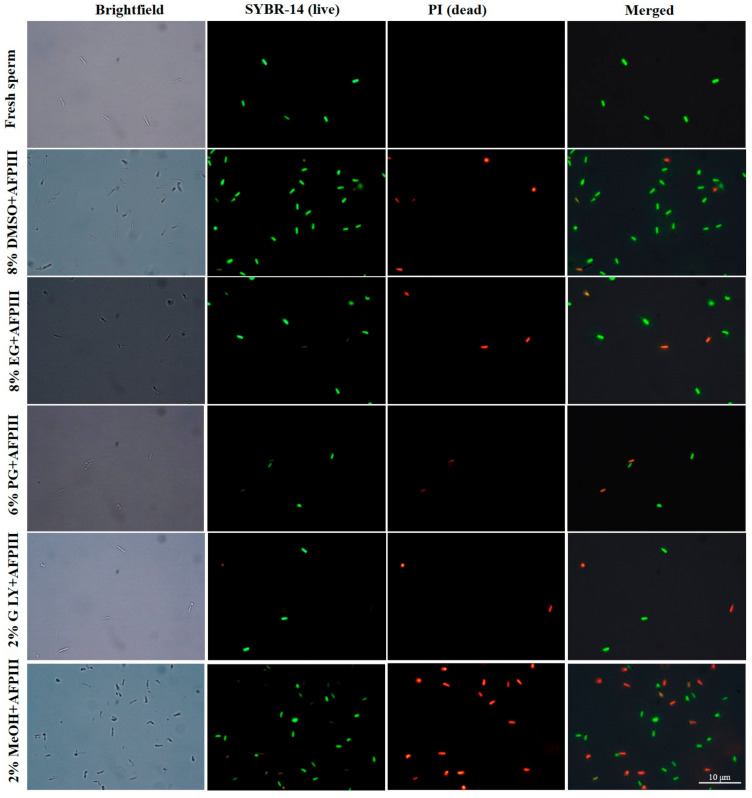
Fluorescent stained photographs for detecting plasma membrane integrity of sperm cryopreserved with AFPIII (1000× magnification).

Sperm cryopreserved using 10 µg/mL AFPIII combined with 8% DMSO showed the highest plasma membrane integrity (67.2 ± 4.0%) (Figure 2 and Figure 3). AFPIII at 10 µg/mL combined with 8% EG (57.1 ± 3.7%) or 2% GLY (56.7 ± 2.6%) did not show significant (*p* > 0.05) differences in the integrity of the plasma membrane.

#### 2.2.2. Acrosome Integrity (AI)

**Figure 4:**
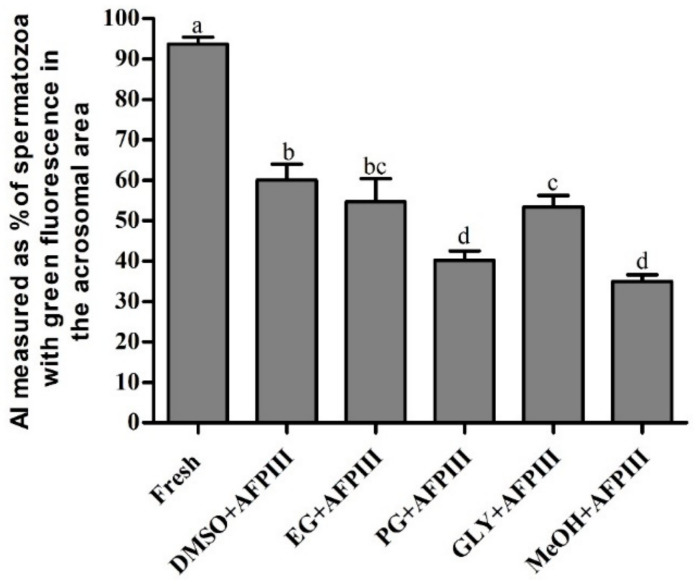
Acrosome integrity (AI) (%) of Pacific abalone sperm cryopreserved with antifreeze protein III (AFPIII). Significantly different levels (*p* < 0.05) are denoted by different letters.

The highest AI value (60.1 ± 3.9%) was found for cryopreserved sperm using 10 µg/mL AFPIII combined with 8% DMSO (Figure 4 and Figure S1). However, this AI value was significantly (*p* < 0.05) lower than the AI of fresh sperm.

#### 2.2.3. Mitochondrial Membrane Potential (MMP)

**Figure 5:**
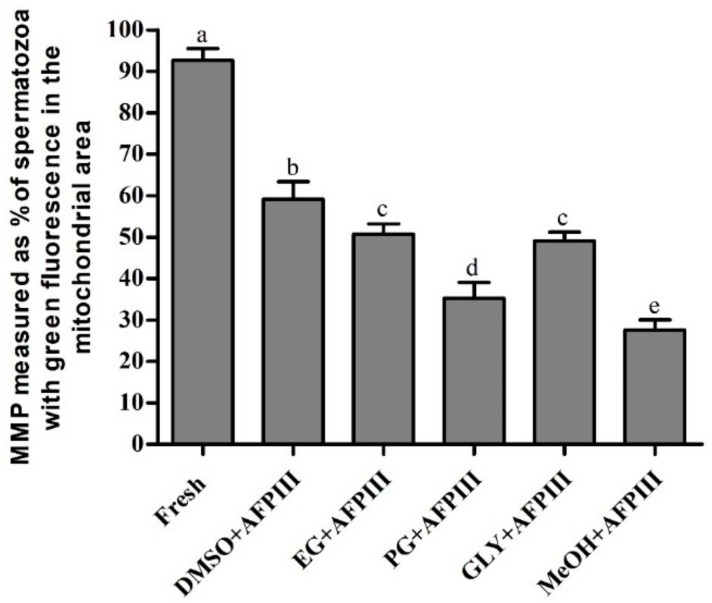
Mitochondrial membrane potential (MMP) (%) of Pacific abalone sperm cryopreserved with antifreeze protein III (AFPIII). Significantly different levels (*p* < 0.05) are denoted by different letters.

Mitochondrial membrane potentials of fresh and cryopreserved sperm are presented in Figure 5 and Figure S2. Sperm cryopreserved with 10 µg/mL AFPIII combined with 8% DMSO had the highest MMP (59.1 ± 4.3%) and sperm with 1 µg/mL AFPIII suspended with 2% MeOH had the lowest MMP (27.63 ± 2.4%). MMP of sperm cryopreserved using 10 µg/mL AFPIII combined with 8% EG or 2% GLY did not show any significant difference (*p* > 0.05). However, fresh sperm showed a significantly (*p* < 0.05) higher MMP value (92.7 ± 2.8%) than all that of all cryopreserved sperm.

### 2.3. DNA Integrity of Fresh and Cryopreserved Sperm

**Figure 6:**
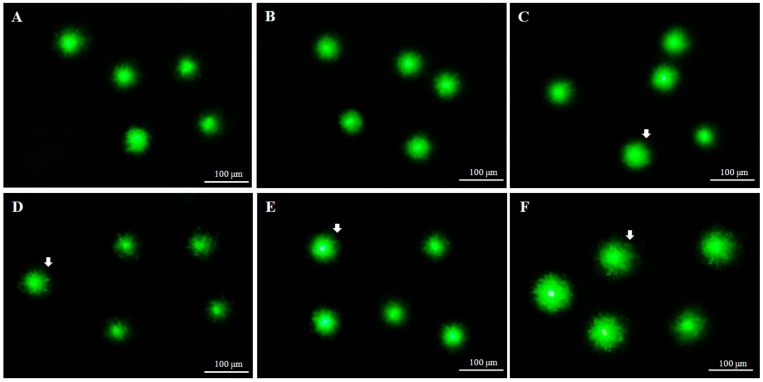
Comet images of fresh and cryopreserved sperm of Pacific abalone. Each comet indicates damaged or undamaged sperm DNA (arrow denotes migration of fragmented DNA from the nuclei of sperm). (**A**) Intact nuclei of fresh sperm. (**B**) Intact nuclei of sperm cryopreserved with 8% DMSO combined with 10 µg/mL AFPIII. (**C**) Intact or slightly damaged nuclei of sperm cryopreserved with 8% EG combined with 10 µg/mL AFPIII. (**D**) Intact or slightly damaged nuclei of sperm cryopreserved with 6% PG combined with 10 µg/mL AFPIII. (**E**) Intact or slightly damaged nuclei of sperm cryopreserved with 2% GLY combined with 10 µg/mL AFPIII. (**F**) Slightly damaged nuclei of sperm cryopreserved with 1 µg/mL 2% MeOH combined with AFPIII.

Comet assay parameters of fresh and cryopreserved sperm using AFPIII are summarized in Table 1. The comet assay did not reveal any significant (*p* > 0.05) difference (Figure 6A,B) between fresh sperm (3.4 ± 2.8% of tail DNA) and sperm cryopreserved using 10 µg/mL AFPIII combined with 8% DMSO (3.6 ± 2.9% of tail DNA). The highest DNA fragmentation (7.6 ± 5.4%) was recorded for sperm cryopreserved using 1 µg/mL AFPIII combined with 2% MeOH (Figure 6F).

### 2.4. Expression Analysis of HSP90 mRNA Transcript

**Figure 7:**
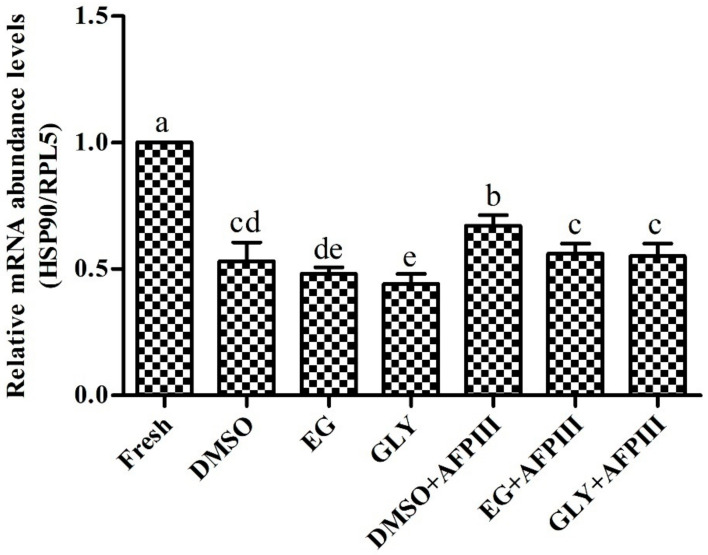
Abundance levels of heat shock protein 90 (HSP90) mRNA in different cryopreserved sperm samples (*n* = 3) of *H. discus hannai*. Significantly different levels (*p* < 0.05) are denoted by different letters.

HSP90 mRNA abundance levels in fresh and cryopreserved sperm samples are shown in Figure 7. Fresh sperm samples showed significantly (*p* < 0.05) higher HSP90 mRNA abundance levels than that of cryopreserved sperm. Sperm cryopreserved using AFPIII combined with a cryoprotectant (CPA) solution (8% DMSO, 8% EG, or 2% GLY) showed significantly (*p* < 0.05) higher HSP90 mRNA abundance levels than those of cryopreserved sperm with control (8% DMSO, 8% EG, or 2% GLY) groups.

### 2.5. Fertility and Hatchability

**Figure 8:**
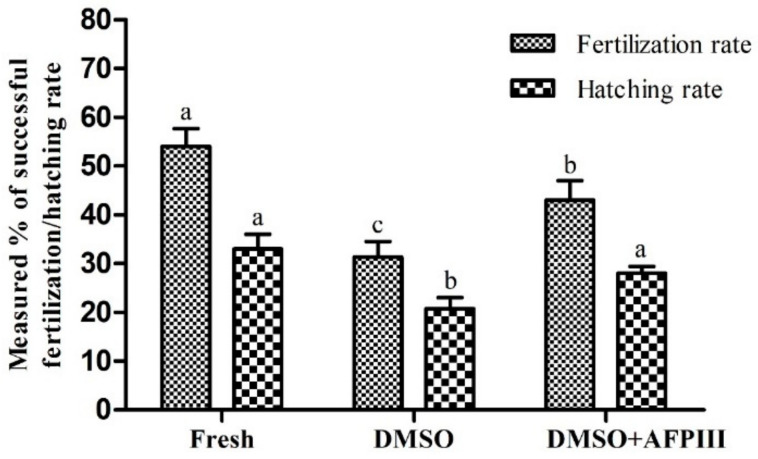
Fertility and hatchability of Pacific abalone sperm cryopreserved using 8% DMSO alone or 8% DMSO in combination with 10 µg/mL AFPIII. Significantly different levels (*p* < 0.05) are denoted by different letters.

Sperm cryopreserved using 10 µg/mL AFPIII combined with 8% DMSO showed a significantly higher fertilization rate (43.0 ± 4.0%) than those preserved with 8% DMSO without AFPIII (31.4% ± 3.1%). Fresh sperm showed a significantly (*p* < 0.05) higher fertilization rate (54.0 ± 3.6%) than that of all cryopreserved sperm (Figure 8). However, hatching rate did not show any significant difference (*p* > 0.05) between fresh sperm (33.0 ± 3.0%) and sperm cryopreserved using 10 µg/mL AFPIII combined with 8% DMSO (28.6 ± 1.4%).

### 2.6. Correlation Among Sperm Quality Parameters

Sperm motility showed strongly positive correlations (Table 2) with fertilization rate (*r* = 0.903; *p* < 0.05), PMI (*r* = 0.994; *p* < 0.01), AI (*r* = 0.990; *p* < 0.01), and MMP (*r* = 0.990; *p* < 0.01). It also showed a moderately positive correlation with hatching rate (*r* = 0.848; *p* < 0.05). However, sperm DNA fragmentation exhibited moderately negative correlations with motility (*r* = −0.858; *p* < 0.01), fertilization rate (*r* = −0.870; *p* < 0.05), hatching rate (*r* = −0.792; *p* < 0.05), PMI (*r* = −0.894; *p* < 0.01), AI (*r* = −0.855; *p* < 0.01), and MMP (*r* = −0.894; *p* < 0.01).

## 3. Discussion

The goal of this study was to optimize the cryopreservation protocol of Pacific abalone spermatozoa. We evaluated the effects of different concentrations of AFPIII on post-thaw sperm quality with special emphasis on HSP90 mRNA abundance levels in cryopreserved sperm of *H. discus hannai*. The current study is the first one using AFPIII for sperm cryopreservation of mollusk species or aquatic invertebrates. AFPIII is a small protein found seasonally in the blood of polar fish. It shows a spherical fold that covers short b-stands with a flat-type ice-binding patch on the surface [35]. Improvement of sperm motility with AFP supplementation is suggested to stem from the possible function of AFP through ion channel blocking and stabilizing transmembrane electrolyte gradients [36]. In the present study, sperm cryopreserved using 10 µg/mL of AFPIII exhibited improved post-thaw sperm motility than that of sperm cryopreserved with only 8% DMSO, 8% EG, 6% PG, or 2% GLY. AFPIII at 1 µg/mL combined with 2% MeOH improved post-thaw sperm motility than that the control (2% MeOH alone). In the present study, addition of AFPIII improved post-thaw sperm motility by about 10% than that previously suggested for CPAs (8% DMSO, 8% EG, 6% PG, 2% GLY, or 2% MeOH) by Kim et al. [2]. Improved motility of sperm cryopreserved using AFPIII has been previously reported for Persian sturgeon [32,37], gilthead seabream [27], bovine [32], and buffalo bull [35,38].

The sperm structure has several compartments bounded by the plasma membrane, the outer acrosomal membrane, and the outer mitochondrial membrane. Furthermore, acrosome integrity (AI), plasma membrane integrity (PMI), and mitochondrial membrane potential (MMP) are quality indicators of sperm [2]. AI is a key indicator of fertility potential. It can be visualized using LYSO-G florescent dye [39]. In the present study, the highest percentage of intact acrosome was found for sperm cryopreserved using 10 µg/mL of AFPIII combined with 8% DMSO. The addition of AFPIII to 8% DMSO improved AI by around 10% over the previously reported findings [2] of cryopreserved sperm using 8% DMSO alone. Such improvement indicates that AFPIII increases the acrosomal resistance to temperature stress.

PMI is a quality indicator of a spermatozoon and is frequently used as a physiological indicator of cryopreserved sperm [14]. Kim et al. [2] have reported PMI values of 54% and 51% for sperm cryopreserved using 8% DMSO and 2% GLY, respectively. The present study revealed improved PMI values by 15% and 6% for sperm cryopreserve using 10 µg/mL AFPIII combined with 8% DMSO and 2% GLY, respectively, compared to PMI values reported by Kim et al. [2]. It has been previously reported that the addition of AFPIII can robustly improve the PMI of cryopreserved sperm of sterlet [9] and seabream [27,28]. Results of the present study indicate that 10 µg/mL AFPIII has the potential to improve the plasma membrane stability of cryopreserved sperm of Pacific abalone. The interaction between AFP and the sperm plasma membrane has been previously reported for different species [9,28].

MMP is a key indicator of mitochondrial activity. It is directly associated with sperm motility and fertility [40,41]. The freeze-thaw process can damage mitochondria and the result is decreased production of ATP [42]. In the present study, sperm cryopreserved using 10 µg/mL AFPIII combined with 8% DMSO resulted in an improved MMP by about 20% over those preserved with 8% DMSO alone without the addition of AFPIII [2]. Improved MMP using AFPIII has also been reported for cryopreserved macaque sperm [41]. The results suggest that the improved motility is most likely the result of less mitochondrial damage, measured as MMP. The present study is the first one to report the effects of AFPIII on MMP of cryopreserved sperm of aquatic animals (fish and shellfish). 

Sperm DNA integrity is an indicator of fertilization capacity and the quality of an embryo. High levels of sperm DNA fragmentation can increase abnormalities of embryos [23]. In the present study, DNA integrity of sperm cryopreserved using 10 µg/mL AFPIII combined with 8% DMSO was similar (*p* > 0.05) to that of fresh sperm. Findings of the present study suggest that the addition of 10 µg/mL AFPIII for cryopreserved sperm of Pacific abalone can reduce DNA fragmentation to a nonsignificant level compared to that of fresh sperm control. Evidence about the use of an antifreeze protein for protecting DNA integrity of cryopreserved fish and shellfish sperm has not been reported yet. However, Kim [43] has reported a similar phenomenon using antifreeze protein for the cryopreserved sperm of pigs.

The present study showed gradually decreased mRNA abundance levels of HSP90 from fresh sperm to sperm cryopreserved with AFPIII or sperm cryopreserved without AFPIII. Sperm cryopreserved with AFPIII exhibited higher mRNA abundance levels of HSP90 than those of control groups (without AFPIII). Present findings indicate that cryopreservation of Pacific abalone spermatozoa has a detrimental effect on the HSP90 mRNA transcript, consistent with previously published results for sperm of oyster [23] and yellow catfish [44]. Reasons for the reduced level of HSP90 mRNA transcript in cryopreserved sperm are due to the susceptibility of HSP90 mRNA transcript to cold stress. The role of HSPs mRNA in sperm fertility has previously been reported in humans, where decreased abundance levels represent decreased fertility [45,46]. Pan et al. [47] reported a lower level of mRNA abundance from vitrified oocyte and early cleavage embryos, which was responsible for a lower cleavage rate.

HSP90 is a molecular chaperone responsible for correct protein folding [48]. HSP90 translation occurs during fertilization [45] and plays an important role in the normal process of myogenesis in embryos [49]. Lower HSP90 function was reported for embryonic developmental defects in zebrafish [50]. The present study suggests that due to cryodamage a lower translation of paternal HSP90 mRNA transcripts occurred, which has a negative impact on correct protein folding. This process may affect the early embryonic development stage by apoptosis and lower cleavage rate.

Fertility is an important indicator of the reproduction success of cryopreserved sperm. The freeze–thaw process of cryopreservation is recognized as a detrimental factor for sperm function and fertility [9]. The goal of the improvement in quality of cryopreserved sperm is to achieve a higher fertilization rate. Sperm cryopreserved using 10 µg/mL AFPIII combined with 8% DMSO had improved fertilization and hatching rates compared with those cryopreserved with control (8% DMSO). Information regarding fertilization experiments of sperm cryopreserved using AFPIII is very limited for fish and shellfish. Xin et al. [9] reported similar fertilization rates of control and AFP used to cryopreserve sperm of *Acipenser ruthenus*. That study also reported similar post-thaw sperm motility regardless of whether AFP was used for cryopreservation. In the present study, improved post-thaw motility might be responsible for the improvement of fertilization and hatching rates. A similar phenomenon has been previously reported for finfish and shellfish sperm [51,52].

The present study showed correlations among sperm quality parameters of Pacific abalone. Sperm DNA fragmentation had negative correlations with all sperm quality parameters. Present findings were consistent with the results of Alcay et al. [53]. Sperm motility exhibited positive relationships with all quality indicators except for DNA fragmentation of sperm. These results were consistent with a previous report for sperm of eastern oyster, *Crassostrea virginica* [54]. The present study focused on how strong the correlation was between each of the quality indicators. The present findings suggest that improved fertility and hatching rate of Pacific abalone sperm might be associated with their higher motility, PMI, AI, and MMP. The present study successfully optimized the sperm cryopreservation protocol of Pacific abalone using 10 µg/mL AFPIII combined with 8% DMSO.

## 4. Materials and Methods

### 4.1. Ethics Statements

Experimental protocols of this study were approved by the Animal Care and Use Committee of Chonnam National University (approval number: CNU IACUC-YS-2020-5). The present study was conducted following Guidelines for the Care and Use of Laboratory Animals of the National Institutes of Health.

### 4.2. Experimental Reagents 

Dimethyl sulfoxide (DMSO), glycerol (GLY), ethylene glycol (EG), propylene glycol (PG), and methanol (MeOH) were obtained from Sigma-Aldrich Pty Ltd. (St. Louis, MO, USA). Antifreeze protein III (AFPIII) was purchased from A/F protein, Inc. (Batch # AFPIII-2016-01). A LIVE/DEAD^®^ sperm viability kit and LysoTracker™ Green DND-26 were purchased from Invitrogen Molecular Probes (Eugene, OR, USA). Rhodamine 123 (Rh 123) and propidium iodide (PI) were purchased from Sigma-Aldrich Pty Ltd. A Comet Assay^®^ Reagent kit was purchased from Trevigen Inc. (Gaithersburg, MD, USA). Phosphate buffer saline (PBS) was obtained from Life technologies Ltd. (Paisley, UK).

### 4.3. Apparatus Arrangement

Styrofoam boxes (Length: 25.0 cm × Width: 25.0 cm × Height: 21.0 cm) with racks placed at heights of 5 cm were used. Liquid nitrogen (LN_2_) up to 5 cm was placed in the Styrofoam box. Rack heights were maintained from the surface of the LN_2_. A digital water bath (J-NBT, JISICO, Seoul, Korea) was used to accomplish the thawing of cryopreserved sperm.

### 4.4. Collection of Experimental Animals

Pacific abalone (*H. discus hannai*) were collected from a commercial abalone hatchery (Tou-Jong soosan, Yeosu, Korea). Three-year-old adult abalone were chosen in May to June of 2020 based on swollen and large gonads with the appearance of a whitish color. A total of 115 abalone were used for conducting the present experiment. Abalone were reared in a cemented tank with continuous supply of seawater and aeration.

### 4.5. Sperm Collection and Handling

Abalone were collected from the rearing tank and dried with paper towels. After drying, abalone were placed in large Petri dishes (150 × 50 mm, SPL Life sciences, Korea) and kept in sunlight for 1 h with the shell facing down and another 20 min with the muscle facing down. Abalone were then gently stripped to collect sperm into the Petri dish. These stripped sperm were then collected in Eppendorf tubes using droppers and immediately refrigerated (4 °C).

### 4.6. Quality Evaluation of Fresh Sperm 

In this study, several parameters including sperm motility, PMI, MMP, and AI were used for quality assessment of fresh sperm. Experiments were accomplished with samples having over 90% motile sperm. An aliquot of 10 µL sperm with a concentration of 9.5 (±0.6) ×10^9^ cells/mL was diluted with 100 µL filtered sea water in an Eppendorf tube. Subsequently, 2 µL of the diluted sperm was added to 100 µL filtered sea water on a glass slide to observe the motility of sperm. The motility of each replication was calculated based on the average value of ten sub-samples. Sperm showing vibrating movement were counted as active sperm [4,55]. PMI, MMP, and AI of sperm were assessed with SYBR14/PI, Rh123/PI, and LYSO-G/PI methods, respectively [2].

### 4.7. Cryopreservation Protocol

**Figure 9:**
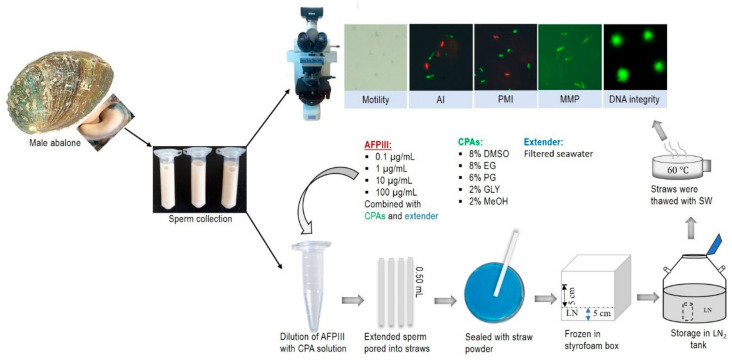
Diagrammatic presentation of the sperm cryopreservation protocol of Pacific abalone, *H. discus hannai*, using an antifreeze protein III (AFPIII).

The following basic cryopreservation procedure (Figure 9) was used based on our previously developed protocol [2]. Briefly, fresh sperm were diluted with filtered seawater (FSW) at a ratio of 1:10. CPA solutions (8% DMSO, 8% EG, 6% PG, 2% GLY, and 2% MeOH) were prepared by dilution with an extender (FSW). Different concentrations of AFPIII were mixed with the cryoprotectant (CPA) solutions. The final solution was prepared by mixing an equal proportion of diluted sperm and the CPA solution. Diluted sperm were equilibrated for 10 min with different concentrations of AFPIII. These equilibrated sperm solutions were then transferred into straws (0.50 mL). Straws (sealed with straw powder) were placed in 5 cm rack heights for 10 min and then immediately submerged into LN_2_ for a minimum of 2 h by tightly closing the Styrofoam box lead. Straws were then collected with large forceps and transferred to a water bath within 4 s for thawing with seawater. The thawing temperature was maintained at 60 °C in the water bath. Post-thaw quality was evaluated under a fluorescence microscope (Nikon eclipse E600).

### 4.8. Effects of AFPIII on Sperm Cryopreservation

AFPIII was separately mixed with 8% DMSO, 8% EG, 6% PG, 2% GLY, or 2% MeOH at a final concentration of 0.1, 1, 10, or 100 µg/mL, respectively. The types of CPA with suitable concentrations (8% DMSO, 8% EG, 6% PG, 2% GLY, and 2% MeOH) were selected based on results of our recently published study [2].

### 4.9. Fluorescent Technique to Assess PMI, AI, and MMP of Cryopreserved Sperm

The fluorescent technique described by Kim et al. [2] was used in this study with slight modifications. The PMI of cryopreserved sperm was assessed using a LIVE/DEAD^®^ sperm viability kit. MMPs of fresh and cryopreserved sperm were evaluated using rhodamine 123 (Rh 123). A LysoTracker^TM^ Green DND-26 (LYSO-G) (Thermo Fisher Scientific) was used for AI evaluation. Propidium iodide (PI) was used to detect damaged sperm cells in each fluorescent evaluation and resulted in a red stain. Sperm samples were diluted with 1X PBS (phosphate buffered saline) at a final volume of 1 mL. To evaluate PMI, 5 µL SYBR 14^®^ was mixed with 1 mL of sample and incubated at 36 °C for 10 min in the dark. After adding 10 µL of PI (2.4 mM), the sample was incubated again at 36 °C for 10 min in the dark. For the AI test, 5 µL of LYSO-G (1 mM) and 10 μL of PI (2.4 mM) were properly mixed with each sample and incubated at 37 °C for 30 min in the dark. To detect MMP, 1 μL of Rh 123 (13 mM) was properly mixed with each sample and incubated at 20 °C for 10 min in the dark. Then, 5 μL of PI (2.4 mM) was added to each sample and incubated at 20 °C for 10 min.

### 4.10. Comet Assay to Detect Sperm DNA Integrity of Fresh and Cryopreserved Sperm

Comet assay was performed according to the method described by Kim et al. [56]. A Comet Assay^®^ kit was used to assess DNA integrity of fresh and cryopreserved sperm. Briefly, sperm were diluted (1 × 10^5^ cells/mL) with chilled 1X PBS and immobilized in agarose gel on comet slides. Comet slides were then immersed in a lysis solution at 4 °C for 1 h. These comet slides were subsequently immersed in a newly prepared unwinding solution for 1 h at 4 °C. Slides were then electrophoresed with a chilled alkaline electrophoresis solution in a comet assay^®^ electrophoresis system for 30 min at 21 V. Vista green dye was used to stain each sample. Comet was immediately visualized and images were captured using a fluorescence microscope (excitation filter 450–490 nm; Nikon eclipse E600). A minimum of 100 sperm cells were analyzed to obtain results of the comet assay. Comet images were analyzed using comet assay IV image analysis software (version 4.3.2, Perceptive Instruments Ltd., UK) to obtain tail length, head length, % DNA in the tail, tail moment, olive tail moment, and extent tail moment.

### 4.11. RNA Extraction and cDNA Synthesis

Fresh and cryopreserved sperm (8% DMSO, 8% DMSO + AFPIII, 8% EG, 8% EG + AFPIII, 2% GLY, 2% GLY + AFPIII) were used to extract total RNA (tRNA). An RNeasy mini kit (Qiagen, Hilden, Germany) was used to isolate tRNA from fresh and cryopreserved sperm samples. Genomic DNA contamination was eliminated using RNase-free DNase (Promega, Madison, WI, USA). Concentrations of tRNAs were then measured with a spectrophotometer (Nanodrop ACTGene ASP-2680, Piscataway, NJ, USA). For cDNA synthesis, tRNA were reverse transcribed using a Superscript^®^ III First-Strand synthesis kit (Invitrogen, Carlsbad, CA, USA) following the manufacturer’s protocol.

### 4.12. Quantitative Real-Time PCR (qRT-PCR) Analysis

A pair of primers (forward: 5′-AACAGTACATCTGGGAGTCG-3′ and reverse: 5′- CCTCCTTGTCTCTTTCCTTCT -3′) designed from a *H. discus hannai* heat shock protein 90 (HSP90, GU014545.1) sequence were used for the qRT-PCR assay to determine mRNA abundance levels of HSP90 in fresh and cryopreserved sperm samples. Ribosomal protein L-5 (RPL-5, GenBank accession no: JX002679.1) (forward: 5′-TGTCCGTTTCACCAACAAGG-3′ and reverse: 5′- AGATGGAATCAAGTTTCAATT-3′) as an internal control was used for normalizing mRNA abundance levels of each of the samples based on its expression stability [57]. A qRT-PCR analysis was performed using a 2× qPCRBIO SyGreen Mix Lo-Rox kit (PCR Biosystems, Ltd., London, UK) as described previously [58,59]. qRT-PCR was performed with the following cycling conditions: a pre-incubation step at 95 °C for 2 min followed by 40 cycles of a three-step amplification at 95 °C for 5 s, 60 °C for 15 s, and 72 °C for 20 s. The relative mRNA abundance levels of HSP90 were quantified using the 2^−ΔΔct^ method [60].

### 4.13. Fertility Test

Good quality and fully matured female abalone (*n* = 30) were chosen based on the greenish color of their swollen gonads. Combined methods of heat treatment and UV-irradiation were used to treat spawning abalone [61,62,63]. Briefly, abalone were exposed to sunlight for one hour with the shell facing down and another 30 min with the shell facing up. Subsequently, they were transferred into inducing buckets and supplied with seawater and a continuous aeration system until spawning. After spawning, egg quality was observed under a microscope (Nikon eclipse E200, Japan). A sperm to egg ratio of 10,000:1 was maintained to check fertilities of fresh and cryopreserved sperm. Sperm cryopreserved using 10 µg/mL AFPIII combined with 8% DMSO or 8% DMSO alone were assessed to determine fertility and hatching success compared to fresh sperm. Water temperature of experimental buckets was maintained between 18 and 20 ℃. Fertilized eggs were washed three times (30 min interval) with FSW (18 to 20 ℃). Fertilization rate (%) and hatching rate (%) were analyzed using the following formulae:

Fertilization rate (%) = Number of fertilized egg/number of eggs counted × 100.(1)

Hatching rate (%) = Number of 14 h hatched larvae/number of fertilized eggs × 100.(2)

### 4.14. Statistical Analysis

Data were analyzed by nonparametric one-way analysis of variance (ANOVA) to generate figures using GraphPad Prism 5.1 software (GraphPad Prism version 5.00 for Windows; GraphPad Software, CA, USA). Duncan’s multiple range test was performed whenever significance was observed. Differences were considered statistically significant at *p* < 0.05. Bivariate correlations were performed following Pearson correlation coefficient analysis with a two-tailed test of significance to determine relationships between post-thaw sperm quality indicators.

## 5. Conclusions

The present study reports an improved sperm cryopreservation technique for Pacific abalone (*H. discus hannai*) using antifreeze protein III (AFPIII). This improvement was due to improved post-thaw motility, AI, PMI, MMP, DNA integrity, fertility, and hatchability. AFPIII at 10 µg/mL combined with 8% DMSO improved post-thaw sperm quality compared to other combinations of CPAs and AFPIII. The comet assay results revealed that the DNA integrity of sperm cryopreserved with 8% DMSO combined with AFPIII was not significantly different from that of fresh sperm. Results of the present study suggest that 10 µg/mL AFPIII combined with 8% DMSO can be used for large scale cryopreservation and fertilization of Pacific abalone sperm for commercial purposes. 

## Figures and Tables

**Table 1:** Comet assay parameters (mean ± standard deviation) for fresh and cryopreserved sperm of *H. discus hannai*.

Parameters	Fresh Sperm	8% DMSO + AFPIII	8% EG + AFPIII	6% PG + AFPIII	2% GLY + AFPIII	2% MeOH + AFPIII
Head length (μ)	50.2 ± 4.3 ^c^	59.4 ± 8.1 ^a^	59.6 ± 6.4 ^a^	57.7 ± 5.7 ^b^	58.5 ± 4.8 ^ab^	60.2 ± 6.2 ^a^
% DNA in head	96.6 ± 2.8 ^a^	96.4 ± 2.9 ^a^	94.9 ± 4.2 ^b^	94.0 ± 4.6 ^b^	94.6 ± 4.3 ^b^	92.4 ± 5.4 ^c^
Tail length (μ)	28.0 ± 5.2 ^c^	29.7 ± 9.3 ^c^	32.4 ± 7.0 ^b^	33.8 ± 6.8 ^ab^	32.0 ± 4.9 ^b^	35.5 ± 13.6 ^a^
% DNA in tail	3.4 ± 2.8 ^c^	3.6 ± 2.9 ^c^	5.1 ± 4.2 ^b^	6.0 ± 4.6 ^b^	5.4 ± 4.3 ^b^	7.6 ± 5.4 ^a^
Tail moment (μ)	0.6 ± 0.5 ^c^	0.8 ± 0.6 ^c^	1.1 ± 0.9 ^b^	1.2 ± 0.9 ^b^	1.1 ± 0.9 ^b^	1.5 ± 1.1 ^a^
Olive tail moment (μ)	2.1 ± 1.3 ^d^	2.7 ± 2.0 ^d^	5.6 ± 3.6 ^c^	7.3 ± 4.1 ^b^	5.8 ± 3.8 ^c^	11.2 ± 6.1 ^a^
Extent tail moment (μ)	94.0 ± 2.6 ^e^	107.1 ± 7.4 ^d^	169.4 ± 6.3 ^c^	204.8 ± 6.12 ^b^	171.4 ± 4.1 ^c^	267.2 ± 14.9 ^a^

Superscripts letters (a–e) indicate significant (*p* < 0.05) differences between fresh and post-thaw sperm.

**Table 2:** Correlation coefficients (*r*) among sperm quality parameters of Pacific abalone, *H. discus hannai*.

	AI (%)	PMI (%)	MMP (%)	DNA Fragmentation (%)	Fertilization (%)	Hatching (%)
Motility (%)	0.990 **	0.994 **	0.990 **	−0.858 **	0.903 *	0.848 *
AI (%)		0.991 **	0.992 **	−0.855 **	0.925 **	0.839 *
PMI (%)			0.994 **	−0.894 **	0.925 **	0.854 *
MMP (%)				−0.885 **	0.935 **	0.853 *
DNA fragmentation (%)					−0.870 *	−0.792 *
Fertilization (%)						0.957 **

** Correlation is significant at *p* < 0.01. * Correlation is significant at *p* < 0.05.

## Data Availability

All data generated in this study are included in this published article and its supplementary information files.

## Supplementary Materials

The following are available online at https://www.mdpi.com/article/10.3390/ijms22083917/s1, Figure S1: Fluorescent stained photographs for detecting acrosome integrity of sperm cryopreserved with AFPIII (1000× magnification). Figure S2: Fluorescent stained photographs for detecting mitochondrial membrane potential of sperm cryopreserved with AFPIII (1000× magnification).

## References

[1] Suleria H.A.R., Masci P.P., Gobe G.C., Osborne S.A. (2017). Therapeutic potential of abalone and status of bioactive molecules: A comprehensive review. Crit. Rev. Food Sci. Nutr..

[2] Kim S.C., Hossen S., Kho K.H. (2020). Cryopreservation of sperm from farmed Pacific abalone, *Haliotis discus hannai*. Cryobiology.

[3] Acosta-Salmón H., Jerry D.R., Southgate P.C. (2007). Effects of cryoprotectant agents and freezing protocol on motility of black-lip pearl oyster (*Pinctada margaritifera* L.) spermatozoa. Cryobiology.

[4] Liu Y., Xu T., Robinson N., Qin J., Li X. (2014). Cryopreservation of sperm in farmed Australian greenlip abalone Haliotis laevigata. Cryobiology.

[5] Mosca F., Madeddu M., Sayed A.A., Zaniboni L., Iaffaldano N., Cerolini S. (2016). Combined effect of permeant and non-permeant cryoprotectants on the quality of frozen/thawed chicken sperm. Cryobiology.

[6] Holt W.V. (2000). Basic aspects of frozen semen storage. Anim. Reprod. Sci..

[7] Swain J.E., Smith G.D., Chian R.C., Quinn P. (2010). Cryoprotectants. Fertility Cryopreservation.

[8] Brum A.M., Sabeur K., Ball B.A. (2008). Apoptotic-like changes in equine spermatozoa separated by density-gradient centrifugation or after cryopreservation. Theriogenology.

[9] Xin M., Tučková V., Rodina M., Kholodnyy V., Dadras H., Boryshpolets S., Shaliutina-Kolešová A., Linhart O. (2018). Effects of antifreeze proteins on cryopreserved sterlet (*Acipenser ruthenus*) sperm motility variables and fertilization capacity. Anim. Reprod. Sci..

[10] Soni Y., Talluri T.R., Kumar A., Ravi S.K., Mehta J.S., Tripathi B.N. (2019). Effects of different concentration and combinations of cryoprotectants on sperm quality, functional integrity in three Indian horse breeds. Cryobiology.

[11] Zilli L., Schiavone R., Zonno V., Storelli C., Vilella S. (2003). Evaluation of DNA damage in *Dicentrarchus labrax* sperm following cryopreservation. Cryobiology.

[12] He S., Woods L.C. (2004). Effects of dimethyl sulfoxide and glycine on cryopreservation induced damage of plasma membranes and mitochondria to striped bass (*Morone saxatilis*) sperm. Cryobiology.

[13] Martin G., Cagnon N., Sabido O., Sion B., Grizard G., Durand P., Levy R. (2007). Kinetics of occurrence of some features of apoptosis during the cryopreservation process of bovine spermatozoa. Hum. Reprod..

[14] Figueroa E., Valdebenito I., Farias J.G. (2016). Technologies used in the study of sperm function in cryopreserved fish spermatozoa. Aquac. Res..

[15] Xin M., Sterba J., Shaliutina-Kolesova A., Dzyuba B., Lieskovska J., Boryshpolets S., Siddique M.A.M., Kholodnyy V., Lebeda I., Linhart O. (2018). Protective role of antifreeze proteins on sterlet (*Acipenser ruthenus*) sperm during cryopreservation. Fish Physiol. Biochem..

[16] Steele E.K., McClure N., Lewis S.E. (2000). Comparison of the effects of two methods of cryopreservation on testicular sperm DNA. Fertil. Steril..

[17] Balamurugan R., Prapaporn W., Munuswamy N. (2019). Sperm activation and effects of cryopreservation on motility, ultrastructure and DNA integrity in grey mullet Mugil cephalus. Aquac. Rep..

[18] Virro M.R., Larson-Cook K.L., Evenson D.P. (2004). Sperm chromatin structure assay (SCSA^®^) parameters are related to fertilization, blastocyst development, and ongoing pregnancy in in vitro fertilization and intracytoplasmic sperm injection cycles. Fertil. Steril..

[19] Balasuriya A., Speyer B., Serhal P., Doshi A., Harper J.C. (2011). Sperm chromatin dispersion test in the assessment of DNA fragmentation and aneuploidy in human spermatozoa. Reprod. Biomed. Online.

[20] Sharma R., Masaki J., Agarwal A. (2013). Sperm DNA fragmentation analysis using the TUNEL assay. Spermatogenesis.

[21] Gwo J.C., Wu C.Y., Chang W.S.P., Cheng H.Y. (2003). Evaluation of damage in Pacific oyster (*Crassostrea gigas*) spermatozoa before and after cryopreservation using comet assay. Cryoletters.

[22] Hezavehei M., Sharafi M., Kouchesfahani H.M., Henkel R., Agarwal A., Esmaeili V., Shahverdi A. (2018). Sperm cryopreservation: A review on current molecular cryobiology and advanced approaches. Reprod. Biomed. Online.

[23] Riesco M.F., Félix F., Matias D., Joaquim S., Suquet M., Cabrita E. (2019). Comparative study on cellular and molecular responses in oyster sperm revealed different susceptibilities to cryopreservation. Aquaculture.

[24] Zhang X.G., Hu S., Han C., Zhu Q.C., Yan G.J., Hu J.H. (2015). Association of heat shock protein 90 with motility of post-thawed sperm in bulls. Cryobiology.

[25] Kasimanickam V., Kasimanickam R., Arangasamy A., Saberivand A., Stevenson J.S., Kastelic J.P. (2012). Association between mRNA abundance of functional sperm function proteins and fertility of Holstein bulls. Theriogenology.

[26] Valcarce D.G., Cartón-García F., Herráez M.P., Robles V. (2013). Effect of cryopreservation on human sperm messenger RNAs crucial for fertilization and early embryo development. Cryobiology.

[27] Beirão J., Zilli L., Vilella S., Cabrita E., Schiavone R., Herráez M.P. (2012). Improving sperm cryopreservation with antifreeze proteins: Effect on gilthead seabream (*Sparus aurata*) plasma membrane lipids. Biol. Reprod..

[28] Zilli L., Beirão J., Schiavone R., Herraez M.P., Gnoni A., Vilella S. (2014). Comparative proteome analysis of cryopreserved flagella and head plasma membrane proteins from sea bream spermatozoa: Effect of antifreeze proteins. PLoS ONE.

[29] Zilli L., Bianchi A., Sabbagh M., Pecoraro L., Schiavone R., Vilella S. (2018). Development of sea bream (*Sparus aurata*) semen vitrification protocols. Theriogenology.

[30] Shaliutina-Kolešová A., Dietrich M., Xian M., Nian R. (2019). Seminal plasma transferrin effects on cryopreserved common carp *Cyprinus carpio* sperm and comparison with bovine serum albumin and antifreeze proteins. Anim. Reprod. Sci..

[31] Abed-Elmdoust A., Farahmand H., Mojazi-Amiri B., Rafiee G., Rahimi R. (2015). Novel droplet vitrification combined with fish antifreeze protein type III enhances cryoprotection of semen in wild endangered Persian sturgeon *Acipenser persicus* (Borodin, 1897). Aquac. Res..

[32] Prathalingam N.S., Holt W.V., Revell S.G., Mirczuk S., Fleck R.A., Watson P.F. (2006). Impact of antifreeze proteins and antifreeze glycoproteins on bovine sperm during freeze-thaw. Theriogenology.

[33] Robles V., Cabrita E., Anel L., Herráez M.P. (2006). Microinjection of the antifreeze protein type III (AFPIII) in turbot (*Scophthalmus maximus*) embryos: Toxicity and protein distribution. Aquaculture.

[34] Kim H.J., Lee J.H., Hur Y.B., Lee C.W., Park S.H., Koo B.W. (2017). Marine antifreeze proteins: Structure, function, and application to cryopreservation as a potential cryoprotectant. Mar. Drugs.

[35] Qadeer S., Khan M.A., Ansari M.S., Rakha B.A., Ejaz R., Husna A.U., Ashiq M., Iqbal R., Ullah N.S. (2014). Akhter. Evaluation of antifreeze protein III for cryopreservation of Nili-Ravi (*Bubalus bubalis*) buffalo bull sperm. Anim. Reprod. Sci..

[36] Rubinsky B., Arav A., Mattioli M., Devries A.L. (1990). The effect of antifreeze glycopeptides on membrane potential changes at hypothermic temperatures. Biochem. Biophys. Res. Commun..

[37] Abed-Elmdoust A., Farahmand H., Mojazi-Amiri B., Rafiee G., Rahimi R. (2017). Metabolic changes in droplet vitrified semen of wild endangered Persian sturgeon *Acipenser persicus* (Borodin, 1997). Cryobiology.

[38] Qadeer S., Khan M.A., Ansari M.S., Rakha B.A., Ejaz R., Iqbal R., Younis M., Ullah N., DeVries A.L., Akhter S. (2015). Efficiency of antifreeze glycoproteins for cryopreservation of Nili-Ravi (*Bubalus bubalis*) buffalo bull sperm. Anim. Reprod. Sci..

[39] Cunha A.T.M., Carvalho J.D.O., Dode M.A.N. (2015). Techniques for sperm evaluation using fluorescent probes. Semin. Ciênc. Agrár..

[40] Agnihotri S.K., Agrawal A.K., Hakim B.A., Vishwakarma A.L., Narender T., Sachan R., Sachdev M. (2016). Mitochondrial membrane potential (MMP) regulates sperm motility. In Vitro Cell. Dev. Biol. Anim..

[41] Wang S., Duan Y., Yan Y., Adar C., Braslavsky I., Chen B., Huang T., Qiu S., Li X., Inglis B.M. (2019). Improvement of sperm cryo-survival of cynomolgus macaque (*Macaca fascicularis*) by commercial egg-yolk-free freezing medium with type III antifreeze protein. Anim. Reprod. Sci..

[42] Watson P.F., Morris G.J., Clarke A. (1981). The effects of cold shock on sperm cell membranes. Effects of Low Temperatures on Biological Membranes.

[43] Kim D.Y. (2016). Evaluation of antifreeze proteins on miniature pig sperm viability, DNA damage, and acrosome status during cryopreservation. J. Emb. Trans..

[44] Bai C., Kang N., Zhao J., Dai J., Gao H., Chen Y., Dong H., Huang C., Dong Q. (2019). Cryopreservation disrupts lipid rafts and heat shock proteins in yellow catfish sperm. Cryobiology.

[45] Li K., Xue Y., Chen A., Jiang Y., Xie H., Shi Q., Zhang S., Ni Y. (2014). Heat shock protein 90 has roles in intracellular calcium homeostasis, protein tyrosine phosphorylation regulation, and progesterone-responsive sperm function in human sperm. PLoS ONE.

[46] Ferlin A., Speltra E., Patassini C., Pati M.A., Garolla A., Caretta N., Foresta C. (2010). Heat shock protein and heat shock factor expression in sperm: Relation to oligozoospermia and varicocele. J. Urol..

[47] Pan Y., Cui Y., Baloch A.R., Fan J., He J., Zhang Y., Zheng H., Li G., Yu S. (2015). Association of heat shock protein 90 with the developmental competence of immature oocytes following Cryotop and solid surface vitrification in yaks (*Bos grunniens*). Cryobiology.

[48] Picard D. (2002). Heat-shock protein 90, a chaperone for folding and regulation. Cell. Mol. Life Sci..

[49] Krone P.H., Sass J.B., Lele Z. (1997). Heat shock protein gene expression during embryonic development of the zebrafish. Cell. Mol. Life Sci..

[50] Yeyati P.L., Bancewicz R.M., Maule J., Van Heyningen V. (2007). Hsp90 selectively modulates phenotype in vertebrate development. PLoS Genet..

[51] Chao N.H., Liao I.C. (2001). Cryopreservation of finfish and shellfish gametes and embryos. Aquaculture.

[52] Matteo D.O., Langellotti A.L., Masullo P., Sansone G. (2009). Cryopreservation of the Mediterranean mussel (*Mytilus galloprovincialis*) spermatozoa. Cryobiology.

[53] Alcay S., Ustuner B., Aktar A., Mulkpinar E., Duman M., Akkasoglu M., Cetinkaya M. (2020). Goat semen cryopreservation with rainbow trout seminal plasma supplemented lecithin-based extenders. Andrologia.

[54] Paniagua-Chávez C.G., Jenkins J., Segovia M., Tiersch T.R. (2006). Assessment of gamete quality for the eastern oyster (*Crassostrea virginica*) by use of fluorescent dyes. Cryobiology.

[55] Gwo J.C., Chen C.W., Cheng H.Y. (2002). Semen cryopreservation of small abalone (*Haliotis diversicolor supertexa*). Theriogenology.

[56] Kim S.C., Hossen S., Kho K.H. (2020). Effects of 3 years of cryopreservation on sperm quality of seven-band grouper, *Epinephelus septemfasciatus*. Aquac. Res..

[57] Wan Q., Whang I., Choi C.Y., Lee J.S., Lee J. (2011). Validation of housekeeping genes as internal controls for studying biomarkers of endocrine-disrupting chemicals in disk abalone by real-time PCR. Comp. Biochem. Physiol. Part C Toxicol. Pharmacol..

[58] Sharker M.R., Hossen S., Nou I.S., Kho K.H. (2020). Characterization of insulin-like growth factor binding protein 7 (igfbp7) and its potential involvement in shell formation and metamorphosis of Pacific abalone, *Haliotis discus hannai*. Int. J. Mol. Sci..

[59] Sharker M.R., Kim S.C., Hossen S., Kho K.H. (2020). Characterization of insulin-like growth factor binding protein-5 (igfbp-5) gene and its potential roles in ontogenesis in the Pacific abalone, *Haliotis discus hannai*. Biology.

[60] Livak K.J., Schmittgen T.D. (2001). Analysis of relative gene expression data using real-time quantitative PCR and the 2^−ΔΔct^ method. Methods.

[61] Morse D.E., Duncan H., Hooker N., Morse A. (1977). Hydrogen peroxide induces spawning in mollusks, with activation of prostaglandin endoperoxide synthetase. Science.

[62] Uki N., Kikuchi S. (1984). Regulation of maturation and spawning of an abalone, *Haliotis* (Gastropoda) by external environmental factors. Aquaculture.

[63] Leighton P., Robinson G., McGowan N. (2008). Abalone hatchery manual. Aquaculture Explained.

