# Supplementary material for: Effects of Antifreeze Protein III on Sperm Cryopreservation of Pacific Abalone, Haliotis discus hannai
Source: Int J Mol Sci. 2021 Apr 10;22(8):3917. doi: 10.3390/ijms22083917 (PMC8069295; doi:10.3390/ijms22083917)
Supplement: Supplementary file 1 [file ijms-22-03917-s001.pdf]

Supplementary File

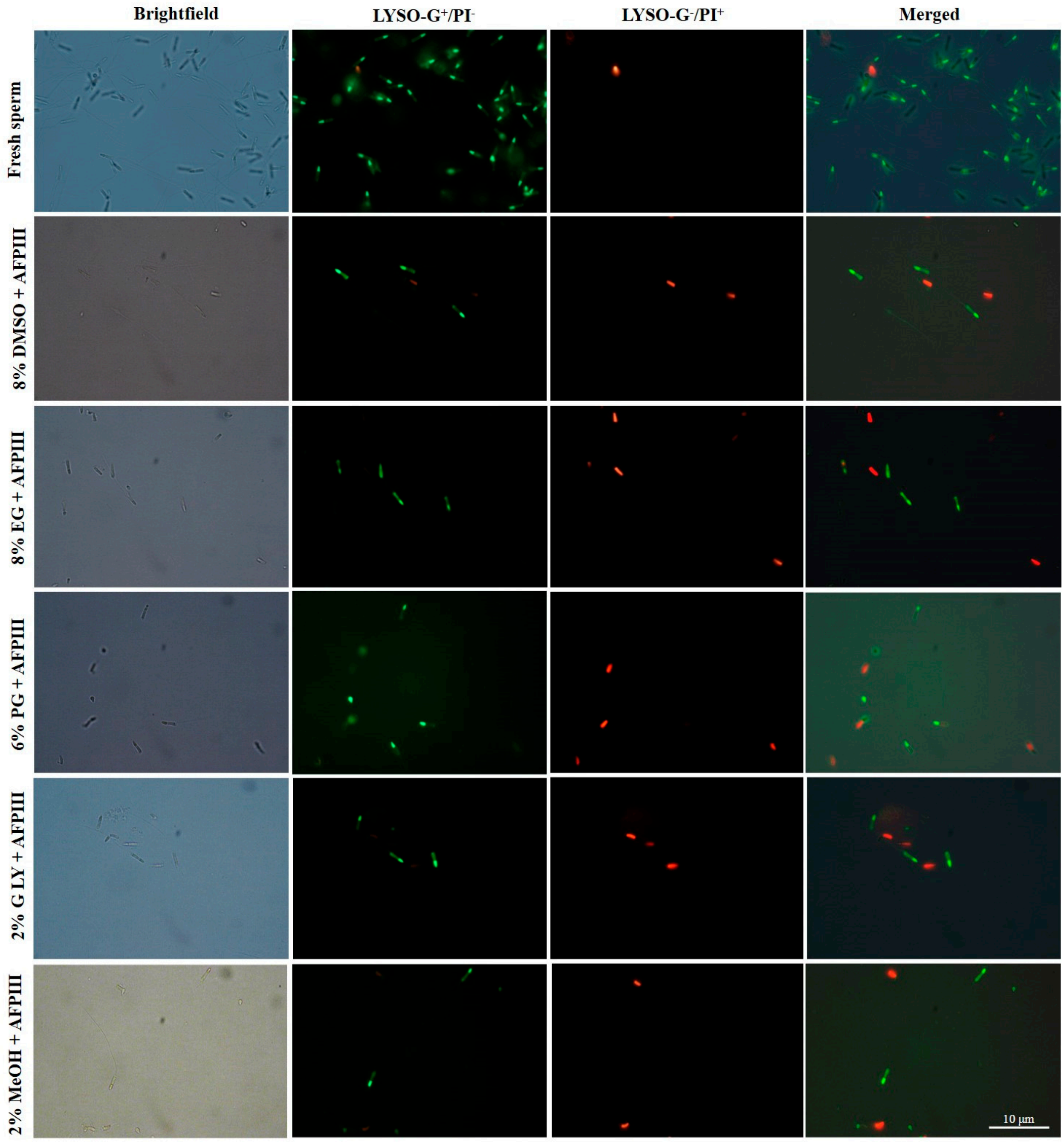

Figure S1. Fluorescent stained photographs for detecting acrosome integrity of sperm cryopreserved with AFPIII (1000x magnification).

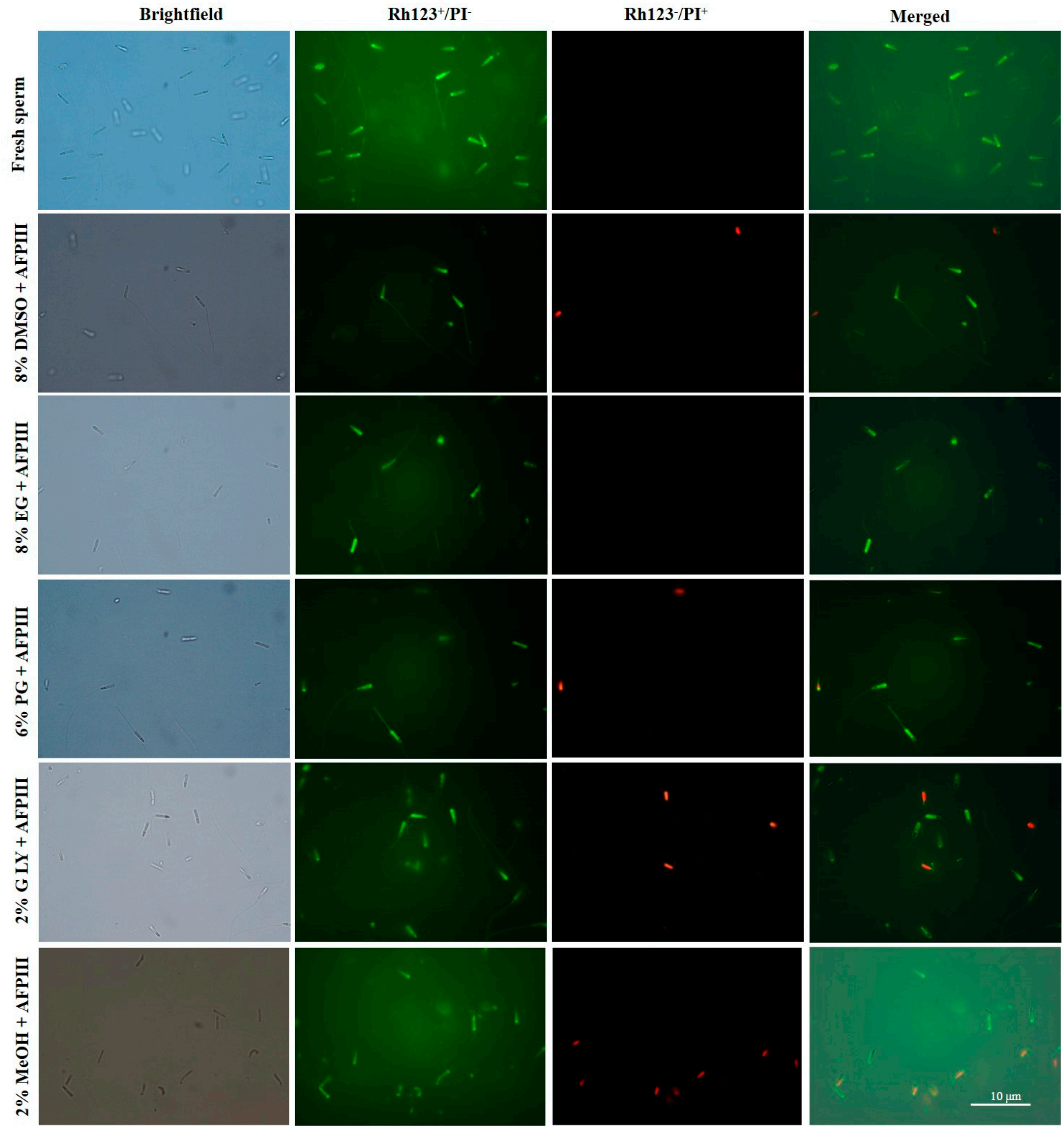

Figure S2. Fluorescent stained photographs for detecting mitochondrial membrane potential of sperm cryopreserved with AFPIII (1000x magnification).
